# Bilayered Fibrin-Based Electrospun-Sprayed Scaffold Loaded with Platelet Lysate Enhances Wound Healing in a Diabetic Mouse Model

**DOI:** 10.3390/nano10112128

**Published:** 2020-10-27

**Authors:** Paola Losi, Tamer Al Kayal, Marianna Buscemi, Ilenia Foffa, Aida Cavallo, Giorgio Soldani

**Affiliations:** 1 Institute of Clinical Physiology, National Research Council, 54100 Massa, Italy; alkayal_tamer@hotmail.com (T.A.K.); marianna.buscemi@ifc.cnr.it (M.B.); ilenia@ifc.cnr.it (I.F.); aida.cavallo@ifc.cnr.it (A.C.); giorgio.soldani@ifc.cnr.it (G.S.); 2 Institute of Life Sciences, Scuola Superiore Sant’Anna, 56127 Pisa, Italy

**Keywords:** platelet lysate, fibrin, electrospinning, spray, phase-inversion, wound healing

## Abstract

The present study examined the effects of a bilayered fibrin/poly(ether)urethane scaffold loaded with platelet lysate by a combination of electrospinning and spray, phase-inversion method for wound healing. In particular, the poly(ether)urethane layer was obtained using by a spray phase-inversion method and the fibrin fibers network were loaded with platelet lysate by electrospinning. The kinetics release and the bioactivity of growth factors released from platelet lysate-scaffold were investigated by ELISA and cell proliferation test using mouse fibroblasts, respectively. The in-vitro experiments demonstrated that a bilayered fibrin/poly(ether)urethane scaffold loaded with platelet lysate provides a sustained release of bioactive platelet-derived growth factors. The effect of a bilayered fibrin/poly(ether)urethane scaffold loaded with platelet lysate on wound healing in diabetic mouse (db/db) was also investigated. The application of the scaffold on full-thickness skin wounds significantly accelerated wound closure at day 14 post-surgery when compared to scaffold without platelet lysates or commercially available polyurethane film, and at the same level of growth factor-loaded scaffold. Histological analysis demonstrated an increased re-epithelialization and collagen deposition in platelet lysate and growth factor loaded scaffolds. The ability of bilayered fibrin/poly(ether)urethane scaffold loaded with platelet lysate to promote in-vivo wound healing suggests its usefulness in clinical treatment of diabetic ulcers.

## 1. Introduction

Wound healing occurs in three sequential phases: hemostasis-inflammation, granulation tissue formation and remodeling. When these phases are absent or do not occur in sequence, we have the formation of chronic ulcers. In the process of wound healing, growth factors are secreted at the wound site by inflammatory cells and provide molecular signals that regulate cellular responses for the wound repair processes such as cell migration, proliferation and differentiation [1]. Growth factor deficiencies, such as platelet-derived growth factor (PDGF) and vascular endothelial growth factor (VEGF) are responsible for chronic wounds [2,3]. This lack of growth factor prevents wound healing and causes the deposition of unorganized extracellular matrix. 

The topical application of growth factors for the clinical therapy of chronic lesions showed conflicting results. This failure could be related to a dilution of growth factor by exudates or inactivation when applied at the wound site. The development of growth factor delivery systems that improve the stability of growth factors and allow the controlled release of factors provide are more effective treatment options [4,5].

Platelet derivates represent a rich source of different growth factors that could address a dynamic and complex process such as wound-healing systems [6]. Therefore, administration of autologous platelet-rich plasma (PRP) gel on the wound sites has been proposed as a strategy to promote the wound-healing cascade and tissue regeneration in chronic wounds, as well as soft tissue ulcerations [7,8].

Platelet lysate (PL) obtained by freeze–thawing cycles of platelet concentrates from peripheral blood has been shown to deliver more growth factors compared to platelet concentrates [9]. Moreover, PL obtained from human peripheral blood promotes in-vitro proliferation, migration and chemotaxis of different cells involved in the wound-healing process [10]. Recently, a combined biological–synthetic scaffold consisting of poly(ether)urethane–polydimethylsiloxane (PEtU–PDMS) material and fibrin, which can deliver bioactive platelet lysate in a controlled and sustained manner and accelerate wound healing in full-thickness skin wounds in a mouse animal model, was described [11].

Recently Wolfe and colleagues demonstrated the feasibility of creating a nanofibrous scaffold from activated PRP to obtain a protein release up to 35 days and cellular colonization throughout the entire scaffold in 3 days [12]. The potential for PRP to be incorporated into electrospun scaffolds of various biological or synthetic materials, such as silk fibroin, poly(glycolic acid) and polycaprolactone, allowing a sustained release of growth factors with positive influence on the bioactivity of the scaffolds, was demonstrated by the same authors [13]. 

In this study a bilayered fibrin/poly(ether)urethane scaffold obtained by a combination of electrospinning and a spray phase-inversion method was used as a platelet lysate growth factor delivery system to evaluate the effect of a bioactive nanostructured layer onto wound healing. The efficacy of the scaffold in promoting dermal tissue regeneration in genetically diabetic (db/db) mice was evaluated.

## 2. Materials and Methods

### 2.1. Ethics Statement

Human blood samples were withdrawn from healthy donors in K3EDTA vacutainers at G. Monasterio Tuscany Foundation, Heart Hospital (Massa, Italy). The hospital is accredited by Regional Health Authority to collect whole blood. The withdrawal method was carried out in accordance with regional guidelines. The approval from institutional review board or ethics committee was thus not necessary. Written informed consent from donors was obtained for using whole blood for research. 

### 2.2. Platelet Lysate Preparation

Platelet-rich plasma (PRP) was obtained by centrifugation at 300 g for 10 min. PRP was centrifuged at 1500 g for 15 min to obtain platelet pellet and platelet poor plasma (PPP). The pellet was resuspended in PPP at 1.1 × 10^9^ platelets/mL. PRP obtained from five different donors was pooled to minimize variations between donors. Finally, PRP was frozen (−80 °C) and thawed (37 °C) to obtain platelet lysate (PL), lyophilized and stored in aliquots at −80 °C until use. A single plasma pool was used either for in-vitro or in-vivo experiments.

### 2.3. Fibrin-Based Electrospun/Sprayed Scaffold Fabrication

The fibrin-based scaffold was fabricated by a combined apparatus which carries out spraying, phase-inversion and electrospinning and is equipped with a metallic rotating cylindrical collector. Three different scaffolds were prepared: a fibrin electrospun/sprayed scaffold (FB scaffold), fibrin-PL electrospun/sprayed scaffold (FB-PL scaffold) and fibrin-growth factor electrospun/sprayed scaffold (fibrin-GF scaffold).

The polymeric support layer was obtained using a biocompatible aromatic poly(ether)urethane (PetU, Estane^®^ 5714F1, Lubrizol, Oevel-Westerlo, Belgium) by a spray, phase-inversion method as previously described [11]. Polyurethane solutions at 0.2% and 2% (*w/v*) were prepared by dissolving polyurethane grain in a solvent mixture of tetrahydrofuran and 1,4-dioxane (1:1). The 0.2% solution was brought near to the precipitation point with the addition of 17% (*v/v*) distilled water as a non-solvent. The support layer was obtained by co-spraying the 2% polyurethane solution and distilled water, while the microporous layer was obtained by co-spraying the 0.2% polyurethane solution and thrombin solution at 25 U/mL in 275 mM CaCl_2_ on a collector of 5 cm in diameter at 88 rpm and a flow rate of 2 mL/min. 

Then, for the bioactive electrospun layer, different solutions were prepared in 1,1,1,3,3,3-heaxafluoro-2-propanol and distilled water (9:1, *v/v*) to obtain: (1) fibrinogen solution (80 mg/mL) for the FB scaffold; (2) fibrinogen/PL solution (50 and 100 mg/mL, respectively) for the FB/PL scaffold; (3) fibrinogen/GF (80 mg/mL, VEGF 10 µg/mL, FGF 25 µg/mL) solution for fibrin-GF scaffold.

Fibrinogen solutions were electrospun onto the poly(ether)urethane support layers. Electrospinning was performed using a needle-to-collector distance of 10 cm, voltage of 22 kV, flow rate of 0.5 mL/h and rotation speed of 125 rpm. The process was prolonged for 5 h. Finally, thrombin solution (25 U/mL) was sprayed on the electrospun layer at a flow rate of 0.5 mL/min for 5 min and the scaffold was incubated at 37 °C for 30 min to allow fibrin polymerization and drying.

Bovine fibrinogen and thrombin were supplied by Merck KGaA (Darmstadt, Germany).

### 2.4. Electrospun/Sprayed Scaffold Morphological Characterization

The surface structures of fibrin-based scaffolds were evaluated by a scanning electron microscope (FlexSEM 1000, Hitachi, Tokyo, Japan). Scaffold samples were completely dry after incubation at 37 °C for 30 min to achieve fibrin polymerization, therefore they were not subjected to other treatments before SEM analysis. SEM microphotographs were taken at 700 and 1000× magnifications with a 5 kV acceleration voltage. The images acquired were analyzed by an open source image processing program (ImageJ). The fibrin nanofibers’ mean diameter was determined by six random measurements for each image. Pore areas was also calculated by a subjective approximation of surface pores in SEM images (areas were calculated by 30 measurements).

### 2.5. Growth Factors Release from Electrospun/Sprayed Scaffold

The platelet-derived growth factor AB (PDGF-AB) and VEGF concentration in PL were evaluated by ELISA (Quantikine; R&D Systems, Minneapolis, MN), according to manufacturer’s instructions.

The release of PDGF-AB and VEFG from the fibrin-PL electrospun/sprayed scaffold was carried out in phosphate buffered saline (PBS). Round samples (1 cm^2^) were incubated in PBS (500 µL/well) under continuous agitation at 37 °C up to 14 days. Daily, for the first seven days and at day 14, the supernatant was collected and an equal volume of PBS was added. The supernatants were stored at −80 °C until growth factor quantification by ELISA.

The cumulative release of PDGF-AB and VEGF were considered and expressed as a percentage of the total amount of loaded growth factor. The experiment was carried out in triplicate.

### 2.6. Bioactivity of Growth Factors Released from Electrospun/Sprayed Scaffold

The bioactivity of PDGF-AB and VEFG released from FB-PL scaffolds was evaluated onto L929 mouse fibroblasts using Transwell methods as previously described [11]. Cell viability after 48 h of incubation was measured by 3-(4,5-dimethylthiazol-2-yl)-2,5-diphenyltetrazolium bromide (MTT) assay.

### 2.7. Wound-Healing Experiments

In total, 48 male diabetic mice (BKS.Cg-m+/+ Lepr, db/db) were supplied by Envigo S.r.l. (Udine, Italy) at 10 weeks and used for the experimentation at 11 weeks. Animal care and experimental protocol were approved by the Italian Ministry of Health via decree n° 905/2018-PR, released on 28th November 2018 according to D. lgs. 26/2014. At each time-point, the animal was weighed and blood glucose levels were measured by a glucometer.

A previously described surgical procedure was employed. Briefly, an 8-mm diameter full-thickness skin wound was created in each mouse. Animals were randomly assigned to following groups (n = 12 for each group): (1) FB scaffold, (2) FB-PL scaffold, (3) fibrin-GF scaffold as a positive control and (4) Mepore^®^ polyurethane film (Mölnlycke Health Care Srl, Gothenburg, Sweden). Mepore polyurethane film is a breathable transparent self-adhesive dressing that does not have any bioactive properties and was used as control.

At 7 days, under anesthesia the scaffolds were removed, the wounds were photographed and fresh scaffolds were applied to the wounds. At 14 days, scaffolds were removed and animals were sacrificed by isoflurane inhalation overdose and wounds were photographed before tissue excision. Tissues from the wounded area were fixed in 10% neutral buffered formalin for histological evaluation.

### 2.8. Determination of Wound Area

The healing area was measured according to the previously described protocol [11]. At 0, 7 and 14 days, wounds were photographed and the wound area was measured by AxioVision Rel. 4.6 software (Carl Zeiss, Oberkochen, Germany). Wound areas were expressed as a percentage of area at day 0: wound area at day X (%) = (wound area at day X/wound area at day 0) per 100.

### 2.9. Histological Assessment of Wound Healing

Samples were embedded in paraffin after dehydration in alcohol series and xilenes and sectioned 7-µm thick perpendicular to the wound. The sections were stained using hematoxylin–eosin (H&E) and Masson’s trichrome with Aniline Blue to evaluate re-epithelialization and collagen deposition. Images were taken with a Zeiss digital camera (AxioCam 105 Color) attached to a Zeiss light microscope (Axio Zoom.V16).

### 2.10. Statistical Analysis

Results are expressed as mean ± standard deviation. Comparison among groups was done with unpaired two-tailed *t*-tests (Stat-View 5.0; SAS Institute, Inc., Cary, NC, USA) and <0.05 was considered significant.

## 3. Results

### 3.1. Electrospun/Sprayed Scaffold Morphological Characterization

**Figure 1:**
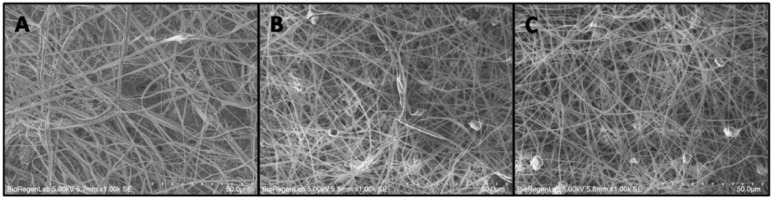
SEM image of (**A**) fibrin electrospun/sprayed (FB) scaffold; (**B**) fibrin-platelet lysate (PL) electrospun/sprayed (FB-PL) scaffold; (**C**) fibrin-growth factor electrospun/sprayed (FB-GF) scaffold. 1000× original magnification (scale bar = 50 µm).

SEM images showed the presence of fibrous structures in all analyzed samples. The FB electrospun scaffold evidenced fibers with a medium diameter of 1.09 ± 0.57 µm (Figure 1A). The addition of PL or growth factors (GF) showed a decrease in the medium diameter of the fibers—0.49 ± 0.25 µm and 0.48 ± 0.23 µm, respectively (Figure 1B,C). In a similar way, the addition of PL or GF showed a decrease in pore areas: 90 ± 51 µm^2^ in FB scaffold, 38 ± 20 µm^2^ in FB-PL scaffold and 45 ± 22 µm^2^ (Figure 1). In all samples the formation of nanofibers with interconnected pores was observed. 

### 3.2. Growth Factors Release from Electrospun/Sprayed Scaffold

**Figure 2:**
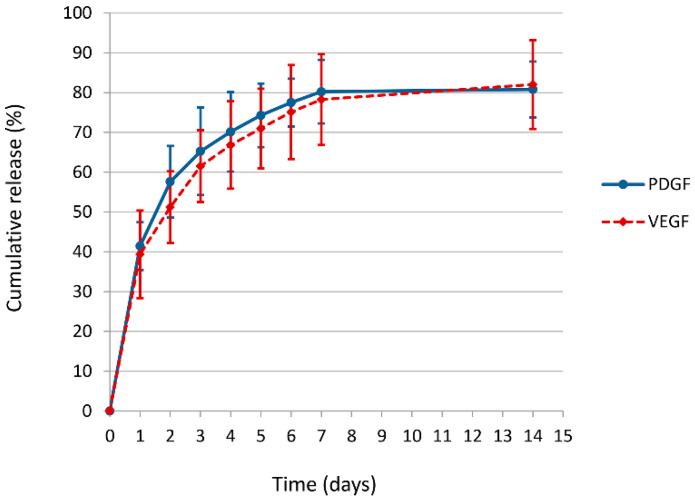
Release kinetics of platelet-derived growth factor AB (PDGF-AB) and vascular endothelial growth factor (VEGF) from FB-PL scaffold. Growth factor amount (*n* = 3) ± SD were reported as percentage of the amount of growth factor incorporated into PL scaffold.

The ELISA results for PDGF-AB and VEGF up to 14 days of incubation are reported in Figure 2. The release kinetics of both PDGF-AB and VEGF showed an initial 40% burst (at day 1), about 80% release at 7 days and no further release at 14 days.

### 3.3. Bioactivity Assay

Cell viability was determined by MTT assay on L929 fibroblasts incubated for 48 h with scaffold samples; PL was added to culture medium, culture medium without serum or complete culture medium. FB-PL scaffold, PL added to culture medium and complete culture medium samples showed no significant difference in cell viability (data not shown). These results demonstrated that growth factors maintain bioactivity after electrospinning.

### 3.4. Wound Area Measurements

**Figure 3:**
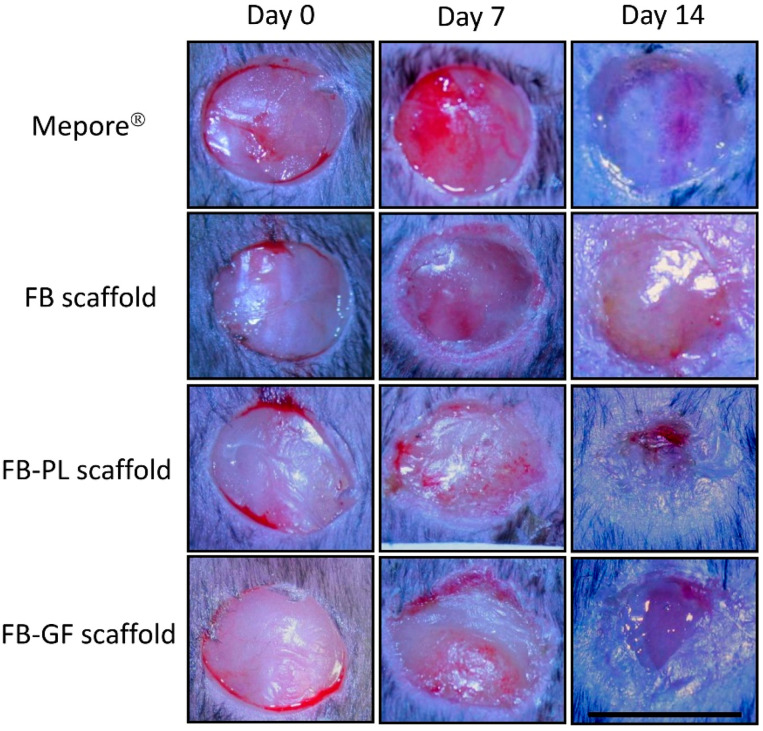
Representative images of lesions treated with FB-PL and FB-GF scaffold in comparison to FB scaffold and Mepore^®^ at day 0, 7 and 14 (scale bar = 1 cm).

**Figure 4:**
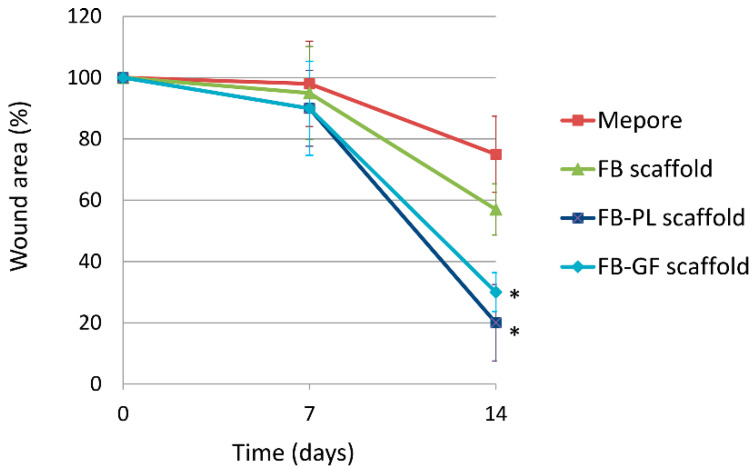
Wound area (expressed as percentage respect to day 0) at day 0, 7 and 14 for each treatment group. Wound closure in FB-PL and FB-GF scaffold is significantly higher respect to FB scaffold and Mepore^®^ groups on day 14 (* *p* < 0.05).

Significant changes of body weight (41 ± 2 g) and blood glucose levels (398 ± 48 mg/dl) were not observed in any mice during the experimental period. Representative wound images obtained for each treatment group at days 0, 7, and 14 post-wounding are shown in Figure 3 and the percentage of wound area in Figure 4. At day 7, no significant differences were found in the wound area of mice treated with all samples. At day 14 after wounding, FB-PL and FB-GF scaffold groups showed a significantly smaller wound area compared to FB scaffold and Mepore^®^ groups.

### 3.5. Histological Assessment of Wound Healing

**Figure 5:**
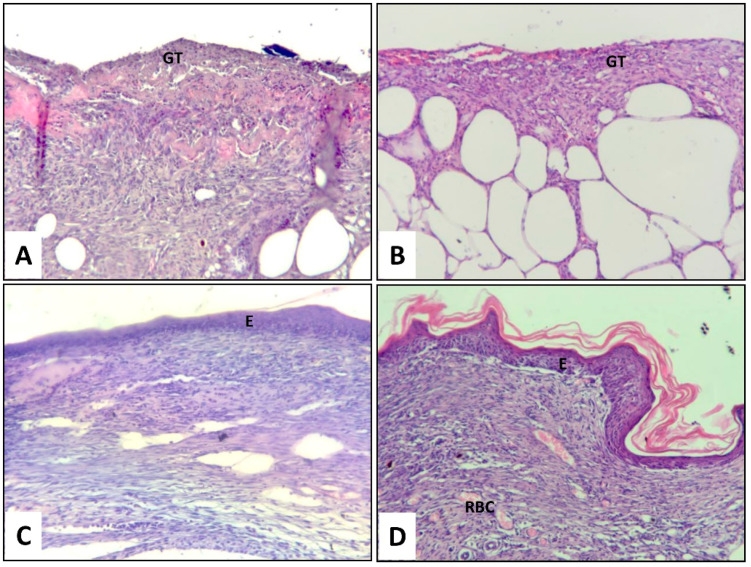
Representative histological tissue sections stained with H&E of wounds treated with (**A**) Mepore^®^ polyurethane film, (**B**) FB scaffold, (**C**) FB-PL scaffold and (**D**) Fibrin-GF scaffold at day 15 after wounding (O.M. 150×). E—epidermis, RBC—red blood cells, GT—granulation tissue.

**Figure 6:**
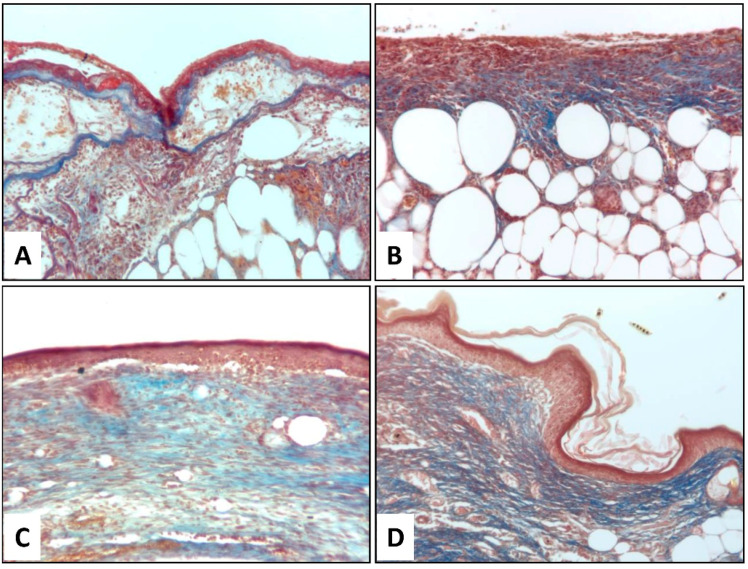
Representative histological sections stained with Masson’s trichrome for evaluation of collagen deposition at day 15 post-wounding (**A**) Mepore^®^ polyurethane film, (**B**) FB scaffold, (**C**) FB-PL scaffold and (**D**) Fibrin-GF scaffold (O.M. 150×). Collagen fibers stained with light Aniline Blue appear blue.

The histological examination of H&E-stained sections on day 14 after wounding revealed that wounds were not fully re-epithelialized in the Mepore^®^ and FB scaffold groups, while complete re-epithelialization was achieved with FB-PL and FB-GF scaffolds. Figure 5 shows images of granulation tissue on day 14 at the center of wounds in all treatment groups. A newly formed epidermal layer was observed only in wounds treated with FB-PL and FB-GF scaffolds. Moreover, the capillaries’ formation by FB-PL and FB-GF was qualitatively higher than both the other scaffolds. Invasive inflammatory cells with no epithelial layer was observed in Mepore^®^ and FB scaffold groups. Masson’s trichrome staining showed the presence of a larger amount of collagen fibers (blue) in the mice treated with FB-PL and FB-GF scaffolds with respect to other groups (Figure 6).

## 4. Discussion

Improper healing of skin wounds is an important problem in medical care. Non-healing wounds are subjected to infections, gangrene, and can results in scars or ulcers. Chronic non-healing ulcers occur in approximately 15–20% of patients with diabetes [14]. With the current treatment for non-healing diabetic foot ulcers, a significant number of patients require hospitalization and in worst cases limb amputation. For these reasons the development of novel treatments to improve the healing of diabetic foot ulcers represents a critical need.

Platelets initiate the wound-healing process by releasing growth factors from their alpha granules, such as PDGF and VEGF [15]. Many researchers have adapted these roles of platelets for cell culture supplements and potential clinical treatment, such as chronic cutaneous ulcer [16].

Recently Notodihardjo and colleagues demonstrated that the stability of growth factors contained in lyophilized human platelet lysate is maintained at 4 °C for up to 9 months. This represents a versatile preservation method for application in clinical practice [17].

In a previous study, the feasibility of creating a nanofibrous scaffold from a PRP was demonstrated. Electrospun scaffolds of PRP proved to be stable for extended periods of time in vitro, exhibited a sustained release of proteins for up to 35 days and promoted rapid cellular infiltration [12]. The same authors successfully incorporated PRP into electrospun scaffolds of various polymers, such as silk fibroin, poly(glycolic acid), and polycaprolactone (PCL). This PRP was released from the electrospun scaffolds in a controlled fashion over a period of 35 days in culture and retained its potential to positively influence the proliferation of adipose-derived stem cells and chemotaxis of macrophages [13].

Other authors showed that hydrophilic nanofibers loaded with platelet-rich plasma produced from chitosan and poly(ethylene oxide) using electrospinning stimulated in-vitro keratinocyte and fibroblast proliferation [18].

Fibrin is an excellent scaffold for tissue-engineering applications because it mimics the extracellular matrix able to support cell adhesion and growth; moreover, fibrin is a suitable scaffold for the local delivery of growth factors and bioactive molecules that drive the healing process [19]. During polymerization, fibrin can be formulated into films, clots, microbeads, nanoconstructs and nanoparticles for drug delivery. Different fibrin formulations offer the possibility to control porosity and degradability and hence to control the drug release [20]. Different studies showed the possibility to electrospin fibrinogen and fibrin to obtain nanostructured scaffold fibrin for tissue-engineering applications [21,22].

Recently a cocoon scaffold loaded with PRP exhibited a better performance compared with the platelet-poor plasma group in full-thickness lesions in New Zealand white rabbits, indicating that the concentration of platelets may be an important factor in wound healing [23].

In our previous work we described a scaffold composed of a combination of poly(ether)urethane-polydimethylsiloxane (PEtU–PDMS) and fibrin loaded with human platelet lysate. The synthetic layer loaded with thrombin was prepared by a spray, phase-inversion process. Then a fibrinogen solution containing platelet lysate was added to form the bioactive fibrin layer. The PL-loaded scaffold showed in vitro sustained growth factors release and accelerated wound healing in a diabetic mouse model of full-thickness skin wounds [11]. 

We prepared platelet lysate from human polled blood samples. The obtained PRP was subjected to a single freeze–thaw cycle to lyse platelets; finally, it was lyophilized to obtain a PL powder. Then, the powder was electrospun with fibrin to obtain a PEtU-fibrin scaffold loaded with PL able to act as local delivery system for bioactive growth factors.

The scaffold described here is composed of a nanostructured FB-PL layer with a bioactive function and a PEtU layer to confer mechanical support of the bioactive layer according to a previous study [24]. The scaffold was manufactured combining two different consolidated techniques: electrospinning and spray, phase-inversion technology. 

The SEM morphological analysis of the FB-PL scaffold showed that PL did not affect fibrin polymerization. SEM analysis evidenced a homogeneous randomly oriented fibrin nanofibers network either onto FB-PL scaffold or onto controls (FB and FB-GF scaffold). The fibers’ mean diameter was similar to fibers in FB-GF scaffolds and smaller with respect to FB scaffold. The appearance of FB network is similar to that described in the literature obtained with fibrinogen 80 mg/mL [25,26].

The in vitro VEGF and PDGF-AB release kinetics were evaluated by ELISA for up to 14 days. The FB-PL scaffold releases a burst of both growth factors on day 1 (about 40%) and a gradual release up to about 80% at day 7. A similar kinetic release was observed in a previous study [11]. To evaluate the bioactivity of the growth factors released from the FB-PL scaffold a cell-based assay was used. The viability of L929 fibroblasts in response to released molecules was evaluated in culture medium without FBS. The L929 cell viability after 48 h incubation with FB-PL scaffold was compared with cell viability of cells incubated with fresh PL, confirming that electrospinning does not affect the bioactivity of PL as demonstrated by Wolfe and colleague [12].

In a diabetic mouse wound-healing model, the local release of platelet lysate from FB-PL scaffold accelerates wound closure, re-epithelialization and collagen deposition with respect to Mepore^®^ and FB scaffold and at the same level of FB-GF scaffold. A previous study demonstrated that a fibrin-based scaffold loaded with pro-angiogenic growth factors induced nearly full-wound healing after 14 days (about 90%) [27].

At day 14 post-injury, higher re-epithelialization and collagen organization were observed in animals treated with FB-PL scaffold respect to animals treated with Mepore^®^ and FB scaffold. These findings are in according to other studies, in which platelet lysate was shown to enhance wound healing in term of collagen deposition and re-epithelialization [17,28]. 

Electrospun nanofibers have been often used as a single or small number growth factor delivery systems due to the high cost of recombinant or purified proteins. The incorporation of PL into electrospun scaffolds as described here could deliver a pool of growth factors in an inexpensive way.

Other authors have described electrospun fibrin scaffolds, however, the employed methods required washing steps for water soluble polymer removal, or a thrombin and CaCl_2_ bath or crosslinking agent use such as glutaraldehyde vapor. For example, Perumcherry and colleagues used poly (vinyl) alcohol (PVA) as an electrospinning driving polymer to electrospun fibrinogen and thrombin solutions by a duploject syringe, after preparation the electrospun scaffold were immersed in PBS to remove PVA polymers [21]. Du and colleagues described a fibrin nanofiber hydrogel obtained for peripheral nerve regeneration electrospinning fibrinogen and poly(ethylene oxide) (PEO) into a rotating collector bath containing CaCl_2_ and thrombin to allow fibrin polymerization. PEO was removed from hydrogel by washing the nanofibers with PBS [29]. McManus used glutaraldehyde vapor fixation, immediately after electrospinning, in a glutaraldehyde vapor chamber for electrospun fibrinogen scaffold crosslinking to regulate scaffold degradation [25]. These methods are not suitable for a drug delivery scaffold preparation since drugs can be released during washing steps or inactivated by glutaraldehyde crosslinking. The scaffold process described in this work does not required any treatment after electrospinning.

Another advantage of the FB-PL scaffold developed in this work using a combined spray/electrospinning technology is to obtain a dehydrated scaffold at the end of the fabrication process. Dehydrated devices decreased the possibility of contamination, facilitated storage, and prolonged platelet lysate growth factors’ storage life. Further research could evaluate the scaffold storage period and temperature that do not affect the bioactivity.
